# ERα PvuII and XbaI polymorphisms in postmenopausal women with posterior tibial tendon dysfunction: a case control study

**DOI:** 10.1186/s13018-018-1020-x

**Published:** 2018-12-11

**Authors:** P. A. Pontin, P. R. B. Nogara, F. C. P. Fonseca, C. Cesar Netto, K. C. Carvalho, J. M. Soares Junior, E. C. Baracat, T. D. Fernandes, N. Maffulli, M. C. L. Santos, A. L. Godoy-Santos

**Affiliations:** 1 Department of Orthopaedic and Traumatology, Hospital das Clinicas HCFMUSP, Faculdade de Medicina, Universidade de São Paulo, São Paulo, SP Brazil; 2 Department of Cell Biology, University Federal of Paraná, Curitiba, PR Brazil; 3 Department of Orthopedic, Hospital for Special Surgery, New York, USA; 4 Department of Gynecology, University of São Paulo, São Paulo, SP Brazil; 5 Department of Orthopaedics, School of Medicine, Surgery and Dentistry, Salerno, Italy; 6 Institute of Science and Technology in Medicine, Keele University School of Medicine, Stoke-on-Trent, UK; 7 Centre for Sports and Exercise Medicine, Queen Mary University of London, Mile End Road, London, E1 4NS UK

**Keywords:** Tendinopathy, Estrogen receptor, Genetic polymorphism, Risk factor

## Abstract

**Background:**

Posterior tibial tendon (PTT) insufficiency is considered as the main cause of adult acquired flat foot and is three times more frequent in females. High estrogen levels exert a positive effect on the overall collagen synthesis in tendons. We have previously demonstrated the association between some genetic single-nucleotide polymorphism (SNP) and tendinopathy. In the present study, we investigated the association of PvuII c454-397T>C (NCBI ID: rs2234693) and XbaI c454-351A>G (NCBI ID: rs9340799) SNPs in estrogen receptor alfa (ER-α) gene with PPT dysfunction.

**Methods:**

A total of 92 female subjects with PTT dysfunction, with histopathological examination of the tendon and magnetic resonance image (MRI) evidence of tendinopathy, were compared to 92 asymptomatic females who presented an intact PPT at MRI for PvuII and XbaI SNPs in the ER-α gene. Genomic DNA was extracted from saliva and genotypes were obtained by polymerase chain reaction restriction fragment length polymorphism.

**Results:**

The analysis of PvuII SNPs showed no significant differences in the frequency of alleles and genotypes between control and PTT dysfunction groups. The XbaI SNPs in the ER-α gene showed significant differences in the frequency of genotypes between control and test groups (*p* = 0.01; OR 95% 1.14 (0.55–2.33).

**Conclusions:**

The XbaI SNP in the ERα gene may contribute to tendinopathy, and the A/A genotype could be a risk factor for PTT tendinopathy in this population. The PvuII SNP studied was not associated with PTT tendinopathy.

## Background

The mechanisms of tendinopathy are complex and involve mechanical stress, degenerative changes in the tendon tissue, and disorganized healing, along with the possible contribution from inflammatory processes. Some studies on the role of estrogens in tendon biology have shown that women are at a higher risk of tendon disorders, with difference according to pre- and post-menopausal status and different phases of the menstrual cycle [1–6].

An increase in estrogens exerts a positive effect on the overall collagen synthesis in tendons, while a decrease in the synthesis of collagen fibers can result from estrogens below physiological levels [7, 8]. Estrogen deficiency may contribute to the age-related decrease in the healing capacity of tendons [9] and may interfere with cell proliferation and matrix synthesis [10, 11].

Posterior tibial tendon (PTT) dysfunction is classically considered to lead to adult acquired flatfoot [12], and several risk factors have been proposed. However, many patients present PTT dysfunction without seemingly any of these risk factors or systemic conditions. It is therefore possible that an interaction between the various intrinsic and extrinsic factors with the genetic make-up of a given individual increases the likelihood of that individual developing tendinopathy. There is an association between of genetic single-nucleotide polymorphisms (SNPs) and tendinopathy [13–16], including PTT dysfunction [17–21].

On the other hand, studies suggest that PTT dysfunction is a consequence and not the origin of adult-acquired flatfoot deformity. The concept of posterior tibial tendon dysfunction as the origin of the deformity is an old paradigm which is being perpetuated in the literature, but we still do not know whether this is the truth [12, 16, 17].

Steroid hormones primarily influence the female reproductive tract, and they are also involved in regulating the metabolism in connective tissues, such as the bone, muscle, and cartilage. The main sources of estrogens are the ovaries and the placenta, but the male testes, the adrenal glands, and several peripheral cells and other tissues, such as osteoblasts, adipocytes, and endothelial cells, also produce small amounts of estrogens [22].

The action of estrogen is predominantly mediated by two classes of estrogen receptor: a rhodopsin-like G protein-coupled receptor, which is located in the endoplasmic reticulum, and two intracellular hormone receptors, namely estrogen receptor alfa and beta (ER-α and ER-β). The latter are members of the nuclear receptor superfamily and are unevenly distributed in many of the estrogen-sensitive tissues.

The ER-α gene is located on chromosome 6q25.1. Two well-studied polymorphisms in the ER-α gene are PvuII c454-397T>C (NCBI ID: rs2234693) and XbaI c454-351A>G (NCBI ID: rs9340799) present on intron 1 and commonly called PvuII and XbaI SNPs. These SNPs have been associated to several estrogen-sensitive traits, including osteoarthritis, scoliosis, osteoporosis, rotator cuff tearing, and breast and prostate cancer [23–28]. In addition, the presence of ER in the human tenocytes of PTT has been demonstrated [29].

Therefore, the purpose of the present study was to investigate the association of PvuII and XbaI SNPs in ER-α gene in female patients with PPT dysfunction.

## Methods

### Study population

This is a case–control cross-sectional study which followed the guidelines of the Declaration of Helsinki. The study protocol was approved by our institutional Ethics committee (10166/2013), and written consent was obtained from each participant.

Participants were patients from the Out-patient Clinics at Department of Orthopedics and Traumatology and Department of Gynecology of the University of São Paulo, Brazil. All were female, aged over 40, and post-menopausal, with at least 12 months of amenorrhea and a follicle-stimulating hormone level > 45 mU/mL [30]. All participants underwent standard clinical examination, including medical history, medication use, personal history of systemic diseases, and infectious or inflammatory diseases. Age, body mass index (BMI), hypertension, hypothyroidism, and age at menopause data were also collected.

Participants were divided into two groups:

- The test group, with 92 post-menopausal females (mean age 59.5 years, range 41–59) who presented PTT dysfunction diagnosed grade II or III (Johnson and Strom Classification System). These patients underwent surgical treatment, and the PTT tendinopathy was confirmed by histopathology (myxoid degeneration associated with multifocal vascular proliferation) and by MRI (T2 image showing intermediate signal intensity and tendon thickening).
- The control group was composed of 92 asymptomatic post-menopausal females (mean age 63.7 years, range 47–62) with no clinical history of PTT disorders and in whom no signal changes of the PTT were evident at MRI. The target population was females in their fourth to sixth decade of life.

All participants were in good general health and did not present any of the following exclusion criteria: BMI greater than 28, diabetes, rheumatic diseases, immunological disorders, liver or kidney disease, and infection or trauma of the foot and ankle. There were no significant differences between the groups in terms of BMI, hypertension, hypothyroidism, and menopause time (*p* > 0.05).

### Genotyping

DNA from epithelial buccal cells was extracted using the procedure previously described [31]. DNA concentration (ng/μL) was estimated by measurements of optical density 260/280 nm ratio greater than 1.9.

The SNPs had previously been identified and reported in the database of the National Center for Biotechnology Information (http://www.ncbi.nlm.nih.gov/SNP/) with minor allele frequencies greater than 0.4.

Genotyping of ER-α PvuII and XbaI SNPs was performed using the polymerase chain reaction restriction fragment length polymorphism (PCR-RFLP) method. The fragment containing ER-α SNPs was amplified using forward 5′-CGTCTACTCCTATGTCTGGT-3′ and reverse 5-CGTGTAGACTGAAGGGCAT-3′ primers. PCR were carried out in a total volume of 50 μL containing 100 ng of genomic DNA, 50 mM KCl, 0.2 mM dNTPs (dATP, dCTP, dGTP, and dTTP), 5 pmol/μL of each primer, 2 mM of MgSO2, and 0.4 unit of Platinum ®Taq DNA Polymerase High Fidelity (Life Biosciences). The PCR condition was set as follows: 94 °C for 6 min, 40 cycles of 94 °C for 30 s, 54 °C for 45 s, and 72 °C for 45 s, and final extension step of 72 °C for 5 min. Then, PCR products were digested with PvuII and XbaI restriction enzymes (Fermentas, Vilnius, Lithuania) and electrophoresed onto 2% agarose. The gel was stained by ethidium bromide and observed under UV light.

### Statistical analysis

Mann–Whitney *U* and Fisher’s exact test were used to determine any significant differences between ages, BMI, hypertension, hypothyroidism, and menopause time of both groups. The chi-square test was applied to compare the frequencies of alleles and genotypes of SNPs in ER-α gene between patients and controls. The program ARLEQUIN (v. 2.0—Schneider et al. [32]) was used to verify the Hardy–Weinberg equilibrium in the population studied.

## Results

All genotype distributions were in Hardy–Weinberg equilibrium. Considering the ER-α PvuII SNPs, there were no significant differences in the frequencies of alleles and genotypes between the control and test groups (Table 1).

**Table 1:** SNPs frequencies of gene ER-α in the control and test groups

SNP	SNPs	Control group	Test group	*p* value	OR^a^ (95% CI)
PvuII c454-397T>C (NCBI ID: rs2234693)	Allele			(Chi-squared)	
	T	51.1 (94)	54.3 (100)	*p* = 0.60	1.13 (0.75–1.71)
	C	48.9 (90)	45.7 (84)		
	Genotype				
	T/T	21.7 (20)	23.9 (22)		
	T/C	58.7 (54)	60.9 (56)	*p* = 0.72	1.13 (0.56–2.25)
	C/C	19.6 (18)	15.2 (14)		
XbaI c454-351A>G (NCBI ID: rs9340799)	Allele	*n *= 184	*n *= 184	(Chi-squared)	
	G	50.5 (93)	43.0 (79)	*p* = 0.17	1.35 (0.90–2.04)
	A	49.5 (91)	57.0 (105)		
	Genotype	*n *= 92	*n *= 92		
	G/G	19.5 (18)	21.7 (20)	*p* = 0.01	1.14 (0.55–2.33)
	A/G	62.0 (57)	42.3 (39)		
	A/A	18.5 (17)	36.0 (33)		

^a^Values are expressed in percentage, with the number of participants (*n*) in parentheses

The ER-α XbaI SNPs showed significant differences in the frequencies of genotypes between the control and test groups. The A/A genotype was found in 36% of the test group and 18.5% of the control group (*p* = 0.01; OR 95% 1.14 (0.55–2.33). In the test group, the A allele was observed with a frequency of 57%, while in the control group, the most frequent genotype was the G allele, but this was not statistically different (*p* = 0.17) (Table 1).

## Discussion

Several genes are highly associated with tendinopathy and tendon rupture and may be useful in constructing a targeted gene panel for patients who have had tendon injuries [16].

There is evidence of an association between SNPs in the estrogen-related receptor beta (ERRβ) and tendon disease. ERRβ exhibits constitutive transcriptional activity and is an orphan receptor that shares significant sequence homology with estrogen receptors ERα and ERβ. Motta et al. [33] identified two SNPs (rs4903399 and rs1676303) in the ERRβ gene which were significantly over-represented in rotator cuff patients compared to controls, while Teerlink et al. [34] showed that the SNP rs17583842 in the same gene was significantly associated with rotator cuff tears. Bonato et al. [35] identified that the SNP rs6574293 in the ERRβ gene was associated with temporomandibular disorders. Also, the SNPs rs10132091 and rs4903399 in the ERRβ gene were associated with comorbidity of temporomandibular disorders and rotator cuff tendinopathy [35].

To our knowledge, this is the first study to analyze the genetic association of SNPs in the ER-α gene in patients with tibialis posterior tendinopathy. These data were derived from 184 participants, providing solid evidence to detect a clinically relevant statistical relationship between SNPs and the condition at hand. All participants were post-menopausal women. The test group was slightly younger that the control group, but there were no significant differences in BMI, hypertension, hypothyroidism, and menopause time, thus minimizing the possible influence of systemic conditions on the pathogenesis of PTT insufficiency.

In the present case–control cross-sectional study, we identified an association between XbaI SNPs in the ER-α gene and PTT insufficiency. The A/A genotype was more prevalent in the test group, and we hypothesize that it may be a risk factor for PTT insufficiency. The A/A genotype may induce greater or faster degradation of the extracellular matrix, which may culminate in tendinopathy of the PTT. The XbaI SNPs lie in intron 1 of the ERα gene, which is part of the A/B domain, the trans-activating factor 1. This domain is a key site to stimulate transcription from certain estrogen-responsive promoters [36]. Among the possible explanations as to how this intronic polymorphism affected PTT dysfunction risk are that intronic changes may have an impact on the expression of receptors by influencing the transcription through alternative splicing of the mRNA transcript [37] or the alteration of another unidentified gene that is adjacent to the ERα gene [38].

Considering the PvuII SNP in ER-α gene, we did not detect significant differences in the frequencies of alleles and genotypes between the control and test groups. However, the effect of this SNP may be masked by SNP in different regions of a gene or other genes that participate in the complex network of mediators from the tendon region.

In addition to that, extensive studies between PvuII and XbaI SNPs in ER-α gene and different pathologies have produced inconsistent results when comparing different ethnic groups. Discrepancies between different studies may result from differences in ethnic background, indicating the probability of inherited susceptibility arising from different genomic ERα SNPs. However, replication studies in other populations as well as functional studies are needed to clarify the complex role of ERα in PTT tendinopathy.

Studies have demonstrated an altered proportion of several types of collagen in tendinopathy; specifically, PTT dysfunction shows increased type III, IV, or V collagen and decreased type I collagen which is diffusely distributed and grossly surrounded by type III fibrils [21, 39]. Since alterations in estrogen levels affect the overall collagen synthesis, the ERα XbaI SNPs might contribute to alteration in collagens in patients with PTT dysfunction.

Further understanding of the biological mechanisms underlying the tendinopathy process is an important prerequisite in developing genomics application. Early genetic identification of individuals at higher risk to develop PTT dysfunction can contribute to appropriate strategies for prevention and treatment of acquired flatfoot in adults.

Studies suggest that posterior tibial tendon dysfunction is a consequence and not the origin of flatfoot deformity. The concept of posterior tibial tendon dysfunction as the origin of the deformity is an old assumption, but it is still unclear whether a cause–effect relationship is present. In some instances, posterior tibial tendon transfer did not produce flatfoot deformity developed over time. This would support the idea that we are not looking at the whole picture and that degenerative changes must be present in multiple soft tissues (including, for example, the spring, interosseous, and subtalar ligaments) for an adult-acquired flatfoot deformity to develop [12, 16, 17, 39].

In conclusion, the XbaI SNPs in the ERα gene could be a risk factor for PTT tendinopathy. The ER-α PvuII SNP does not appear to be associated with PTT tendinopathy. Larger studies in other ethnic group may clarify the clinical impact of such findings.

## Abbreviations

BMI: Body mass index
ER-α and ER-β: Estrogen receptor alfa and beta
MRI: Magnetic resonance image
PCR-RFLP: Polymerase chain reaction restriction fragment length polymorphism
PTT: Posterior tibial tendon
SNP: Single-nucleotide polymorphism
